# Kardar–Parisi–Zhang roughening associated with nucleation-limited steady crystal growth

**DOI:** 10.1038/s41598-023-43002-3

**Published:** 2023-09-26

**Authors:** Noriko Akutsu

**Affiliations:** Faculty of Engineering, Osaka Electro-Communication University, Hatsu-cho, Neyagawa, Osaka 572-8530 Japan

**Keywords:** Nanoscale materials, Scaling laws, Phase transitions and critical phenomena, Applied physics, Nonlinear phenomena

## Abstract

The roughness of crystal surfaces and the shape of crystals play important roles in multiscale phenomena. For example, the roughness of the crystal surface affects the frictional and optical properties of materials such as ice or silica. Theoretical studies on crystal surfaces based on the symmetry principle proposed that the growing surfaces of crystal growth could be classified in the universal class of Kardar–Parisi–Zhang (KPZ), but experiments rarely observe KPZ properties. To fill this the gap, extensive numerical calculations of the crystal growth rates and the surface roughness (surface width) have been performed for a nanoscale lattice model using the Monte Carlo method. The results indicate that a (001) surface is smooth within the single nucleation growth region. In contrast, the same surface is atomically smooth but thermodynamically rough in the poly-nucleation growth region in conjunction with a KPZ roughness exponent. Inclined surfaces are known to become Berezinskii–Kosterlitz–Thouless (BKT) rough surfaces both at and near equilibrium. The two types of steps associated with the (001) and (111) terraces were found to induce KPZ surface roughness, while the interplay between steps and multilayered islands promoted BKT roughness.

## Introduction

Surface roughness is a complex phenomenon to analyze even in the case that the length scale is limited to less than 2 μm^1^. This topic is also complicated by the possibility of two types of roughness: atomic^2^ and thermodynamic. Molecular dynamics (MD) simulations^3^ have demonstrated that a crystal surface becomes diffuse over several surface layers, often accompanied by lattice distortions. Atomic roughness also tends to increase the surface growth velocity, *V*. In contrast, an atomically smooth surface is sharp on the atomic scale.

Thermodynamic roughness^4,5^ can be defined based on the criteria

1 $$\begin{aligned} \text{Rough\, surface:} \ W \rightarrow \infty \ \text{as } \ L \rightarrow \infty ; \quad \text{Smooth\,\,surface:} \ W \rightarrow \mathrm{const.} \ \text{as } \ L \rightarrow \infty , \end{aligned}$$

where *L* is the linear size of the system and $W=W(L,t)=\sqrt{[h( {\vec{x}} ,t)-\langle h( {\vec{x}} ,t)\rangle ]^2}$, in which $h({\vec{x}},t)$ is the height of the surface at site ${\vec{x}}$ at time *t*. At equilibrium, there will be a thermal roughening transition temperature, $T_\text{R}$, for a two-dimensional (2D) surface in a 3D system. For a given temperature, $T<T_\text{R}$, the surface will be smooth based on Eq. (1) while, at $T_\text{R}<T$, the surface will be rough with $W^2$ diverging logarithmically from *L*. The latter is characteristic behavior linked to the Berezinskii–Kosterlitz–Thouless (BKT) universality class^6,7^. This thermodynamic roughening transition is directly connected to a shape transition occurring at equilibrium and referred to as the faceting transition^8–11^. This phenomenon is associated with the equilibrium crystal shape (ECS), which is the shape of a crystallite having the least total surface free energy.

It should also be noted that an atomically rough surface is different from a thermodynamically rough surface. Examples of surfaces that are atomically rough but thermodynamically smooth include the facet surfaces of a $^4$He crystal in superfluid He^12,13^ and of Pb^14^, Ag$_2$S^15,16^ and Ag$_2$Se^15^. An example of an atomically smooth but thermodynamically rough surface is the inclined (stepped) surface of a lattice model^17–19^. Here, the “inclined surface” is a surface tilted from a singular surface which forms a cusp singularity on the Wulff figure (the polar graph of the surface tension). The singular surface then forms a facet plane on the ECS at lower temperatures than $T_\text{R}$ of the singular surface^8–11^.

In the case of kinetic roughening, various types have also been reported. Based on studies of crystal growth, kinetically rough surfaces are thought to grow via an adhesive process^20–27^. In this case, the surface grows linearly as a response to the driving force for crystal growth, $\Delta \mu$, defined as $\Delta \mu = \mu _\text{ambient}- \mu _\text{crystal}$, where $\mu _\text{crystal}$ is the bulk chemical potential of the crystal and $\mu _\text{ambient}$ is the bulk chemical potential of the ambient phase. The kinetic roughening point, $\Delta \mu _c$, was studied by Van Veenendaal et al.^22^, considering the experimental definition of kinetic roughening, and Cuppen et al.^23^, considering a non-equilibrium definition of kinetic roughening that also includes MC simulations for the nearest neighbor (nn) Kossel solid-on-solid (SOS) model. For so-called classical criteria^22^, $\Delta \mu _c$ is calculated based on the relationship $\pi \gamma _s^2/(3k_\text{B}T\Delta \mu _c) \sim 1$^25^, where $\gamma _s$ is the step tension, which is the step free energy per unit length.

Meanwhile, in the field of statistical mechanics, the term kinetic roughening is used to refer to Kardar–Parisi–Zhang (KPZ) roughening^28^. For fluctuating surfaces (or interfaces), the Family–Vicsek scaling relationship^29–35^ has been widely used to describe the self-affine surface. The Family–Vicsek scaling relationship for a surface can be expressed as

2 $$\begin{aligned} W(L,t){} & {} \sim L^\alpha f(L^{-z}t), \ z=\alpha /\beta , \nonumber \\ W(L,t){} & {} \sim L^\alpha \ \text{ as } \ t \rightarrow \infty , \end{aligned}$$

where $\alpha$, $\beta$ and *z* are the roughness, growth and dynamic exponents, respectively. In the non-equilibrium steady state, the surface width is characterized by the roughness exponent $\alpha$. Based on the symmetry principle, a surface growth equation including a nonlinear term obtained from the KPZ model^28^ can be derived as

3 $$\begin{aligned} \frac{\partial h_t}{\partial t}= v_0 + \nu \nabla ^2 h_t + \frac{\lambda }{2} (\nabla h_t )^2 + \eta _t \end{aligned}$$

where $h_t$ is the surface height at time *t*, $v_0$ is the constant surface velocity, $\nu >0$ is a coefficient related to surface tension, $\lambda$ is a coefficient for the nonlinear term and $\eta _t$ is white noise in space and time. In the case of a 2D surface in a 3D system, the values of these exponents are predicted to be $\alpha =0.3869$, $\beta = 0.2398$ and $z=1.6131$^34,35^ (KPZ-rough surface). The experimentally determined values of various systems such as directed polymers are known to agree with these exponents, indicating that these systems belong to the KPZ universality class. However, in the case of crystal growth, the observed roughness exponents tend to differ from those predicted by the KPZ model^30,35,36^, with the exception of several special surface systems^37,38^.

In our previous work^39^, we found crossover phenomena between BKT rough and KPZ rough surfaces at $\Delta \mu _{cr}$ for inclined surfaces using the Monte Carlo method based on the nn restricted solid-on-solid (RSOS) model on a square lattice. Here, the term “restricted” indicates that the surface height difference between nearest neighbor sites is limited to $\{0, \pm 1\}$. In the work, thermally excited structures such as adatoms, adholes, islands or negative islands (clusters of adholes) on terraces are found to cause the crossover phenomena. These thermally excited structures are thought to be irrelevant and they only renormalize the step tension for the thermal roughening transition at equilibrium. However, several Monte Carlo results on the surface width *W* suggested a complex surface slope dependence of *W*.

The aim of the present work is to clarify what makes the growing crystal surface KPZ rough using the RSOS model with and without surface steps. In addition, we obtain the detailed surface slope dependence of *W* explicitly. For this purpose, extensive numerical data on growth rate and surface roughness of planar or inclined surfaces were collected using the Monte Carlo method for a non-equilibrium steady state.

The RSOS model is more restricted than the Kossel SOS model studied by Van Veenendaal et al.^22^ or the absolute SOS (ASOS) model^4,40^. However, the model has a special characteristic in that an almost ideal terrace-step-kink (TSK) model^41,42^ is realized^39,43^ around a (111) surface due to the RSOS restriction. In the ideal TSK model, thermally excited structures are prohibited on the terrace surface^44,45^.

The RSOS model is a more microscopic model than the models used in phase field calculations^46^, whereas it is a coarse grained model for the purposes of first principles quantum mechanical calculations^47^.

It should also be noted that the RSOS model is equivalent to a 19-vertex model^48,49^ and, because the latter represents a non-integrable system, the RSOS model cannot be solved exactly using the Bethe ansatz approach^50^. This is one of the reasons why the present work chose to study the RSOS model numerically.

At equilibrium, the RSOS model employed in the present work is equivalent to that previously used to determine roughness exponents by Amar and Family^51,52^. It is also important to note that a model used in the field of nonlinear dynamics with the restriction of the height difference being an integer is also sometimes referred to as the RSOS model but is known as the ASOS model in the field of roughening transition studies^4,40^.

During the present work, surface diffusion, volume diffusion and elastic effects were not taken into consideration.

## Model and calculations

### RSOS model

The surface energy of a surface with an orientation close to (001) exhibiting (001) terrace roughness can be expressed by the discrete Hamiltonian^39^

4 $$\begin{aligned} \mathscr{H}_\text{RSOS} = \mathscr{N}\varepsilon _\text{surf}+ \sum _{n,m} \varepsilon [ |h(n+1,m)-h(n,m)| +|h(n,m+1)-h(n,m)|] - \sum _{n,m} \Delta \mu \ h(n,m), \end{aligned}$$

where *h*(*n*, *m*) is the surface height at site (*n*, *m*) on a square lattice, ${\mathscr{N}}$ is the total number of lattice points, $\varepsilon _\text{surf}$ is the surface energy per unit cell on the planar (001) surface and $\varepsilon$ is the microscopic ledge energy associated with nearest neighbor (nn) interactions. The summation with respect to (*n*, *m*) is over all sites on the square lattice. The RSOS condition, meaning that the height difference between nearest neighbor sites is restricted to $\{ 0, \pm 1\}$, is required implicitly. In this equation, $\Delta \mu$ is the driving force for crystal growth. To exclude diffusion effects, the ambient phase is assumed to be uniform. In the nanometer length scale near equilibrium, this assumption can be realized. In the case that the ambient phase is an ideal solution, $\Delta \mu = k_\text{B}T\ln C/C_\text{eq}$^53^, where $k_\text{B}$ is the Boltzmann constant, *T* is temperature, *C* is the concentration of the solute and $C_\text{eq}$ is the concentration of the solute at saturation. If the ambient phase is an ideal gas, $\Delta \mu = k_\text{B}T\ln P/P_\text{eq}$^54^, where *P* is the gas pressure and $P_\text{eq}$ is the gas pressure at equilibrium. $\varepsilon$ and $\Delta \mu$ in the present model correspond to $\phi$ and $\Delta \mu$ in Van Veenendaal et al.’s work^22^.

Since the RSOS model is a coarse grained model used for the purpose of first principles quantum mechanical calculations, $\varepsilon _\text{surf}$ and $\varepsilon$ relate to the surface free energy in the atomic model include the entropy for lattice vibrations and distortions^47^. Thus, these variables are affected by temperature. However, the present work assumes constant values for $\varepsilon _\text{surf}$ and $\varepsilon$ in all calculations.

### Monte Carlo calculations

In this work, the surface configuration was updated using the Metropolis algorithm and the energy difference, $\Delta E$, was calculated based on Eq. (4). The first $2 \times 10^8$ Monte Carlo steps per site (MCS/site) were ignored and each quantity was averaged over the subsequent $2 \times 10^8$ MCS/site. The surface slope *p* and the surface growth velocity *V* were calculated as macroscopic variables, such as

5 $$\begin{aligned} p=N_\text{step}a/L, \quad V=(\langle h(t+\tilde{\tau })\rangle -\langle h(t)\rangle )/\tilde{\tau }, \end{aligned}$$

where $N_\text{step}$ is the number of steps, which was fixed during the simulation, $a=1$ is a lattice constant and $\tilde{\tau }$ is set to $2 \times 10^8$ MCS/site.

When considering an inclined surface, the squared surface width was calculated as

6 $$\begin{aligned} gW^2 =\langle \langle [h(\tilde{x}, \tilde{y}, t)- \langle h(\tilde{x}, t)\rangle _{\tilde{y}}]^2\rangle _{\tilde{y}} \rangle _{\tilde{x}}, \end{aligned}$$

where *W* is a surface width normal to the inclined surface, *g* is a geometrical factor defined as $1+p_x^2 +p_y^2$ with $p_x=\partial \langle h \rangle /\partial x$ and $p_y=\partial \langle h \rangle /\partial y$^55^, $\tilde{x}$ and $\tilde{y}$ are the [110] and $[ \bar{1}10 ]$ directions, respectively, and $\langle \cdot \rangle _{\tilde{y}}$ and $\langle \cdot \rangle _{\tilde{x}}$ are the averages over the $\tilde{y}$ and $\tilde{x}$ directions.

Periodic boundary conditions were adopted in the vertical ($[\bar{1}10]$) direction. In the horizontal ([110]) direction, periodic boundary conditions were adopted while also adding the number of steps, $N_\text{step}$.

Crystal growth proceeds by the attachment/detachment of specific units. As such, the number of units in the crystal does not have to be conserved during the process, making this a non-conserved system. The present work also did not include unit exchange on the surface, meaning that surface diffusion was neglected. At equilibrium, the unit attachment rate will equal the detachment rate. The attachment rate automatically increases whereas the detachment rate decreases as $\Delta \mu$ increases.

**Figure 1:**
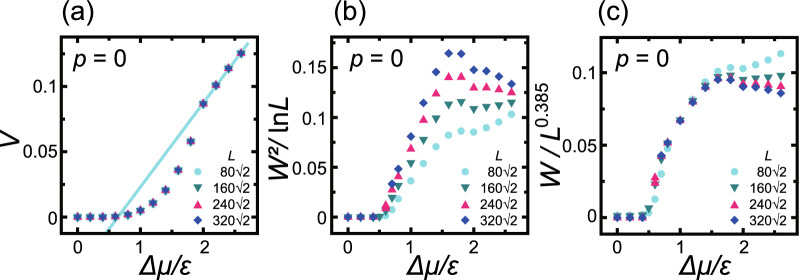
Surface growth velocity and scaled surface widths at the (001) surface ($p=0$) as functions of $\Delta \mu$. (**a**) Surface growth velocity with unit of $a/\tau$, where *a* ($=1$) is the unit height and $\tau$ is the time interval for 1 MCS/site. Line: $V= 0.0643\Delta \mu /\varepsilon -0.0412$. (**b**) The squared surface width, $W^2=\langle [h({\vec{x}} )-\langle h \rangle ]^2\rangle$, scaled by the logarithm of the system size, *L*. (**c**) The surface width scaled by $L^{\alpha }$ with the roughness exponent $\alpha \approx 0.385$ determined from the KPZ model. $k_\text{B}T/\varepsilon =0.4$.

**Figure 2:**
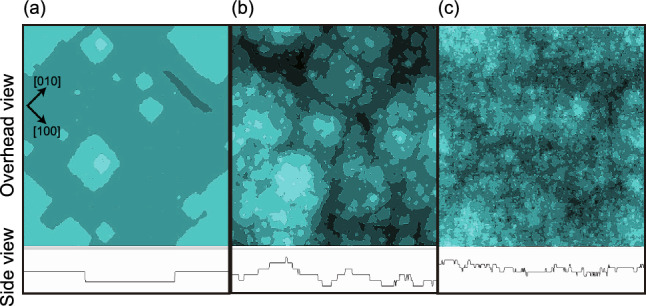
Images of simulated $p=0$ surfaces. Upper figures: overhead views. Lower figures: side views showing the height along the lower perimeters of the upper figures for$\Delta \mu /\varepsilon =$ (**a**) 0.8, (**b**) 1.4 and (**c**) 2.6. $k_\text{B}T/\varepsilon =0.4$. $L = 320 \times \sqrt{2}$. $\Delta \mu _{cr}/\varepsilon = 0.3$^39^. To better indicate the shapes of the steps on the crystal surfaces, the surface height is represented by 10 degrees of brightness, with a brighter color corresponding to a greater height.

## Results

### KPZ roughening on a (001) Surface

#### Monte Carlo results

Figure 1 presents the Monte Carlo results for the surface growth velocity, *V*, and the scaled surface width, *W*, with regard to the (001) surface. Figure 1a indicates that the surface grows exponentially with respect to $\Delta \mu$ during the 2D nucleation process for $\Delta \mu /\varepsilon <2.0$. In contrast, for $2.0 \le \Delta \mu$, the surface grows linearly via an adhesive growth process. Because the temperature value of $k_\text{B}T/\varepsilon = 0.4$ is far less than the thermal roughening temperature of $k_\text{B}T_\text{R}/\varepsilon = 1.578$^48, 56^, the (001) surface is atomically and thermodynamically smooth at equilibrium. It should be noted that the 2D critical nucleus sizes on the (001) surface were determined to be 2*a* and *a* for $\Delta \mu /\varepsilon =1$ and 2, respectively, assuming that each nucleus was a square. In addition, the 2D critical nucleus sizes at the edges of the straight (01) steps were less than *a* for $1< \Delta \mu /\varepsilon$. In these processes, an atom (that is, the growth unit) attached at the edges of the steps associated with an island will increase the island’s size on average in the case of $1<\Delta \mu /\varepsilon$. The attachment of an adatom to the (001) surface, which is also regarded as an island, will increase its size on average for $2<\Delta \mu /\varepsilon$.

Figure 1b,c provide the scaled surface width data. Near equilibrium and for $\Delta \mu /\varepsilon <0.55$, $W = 0$ and the surface is atomically and thermodynamically smooth. However, in the region defined by $0.55 \le \Delta \mu /\varepsilon <2.0$, the surface width, *W*, increases as the system size, *L*, increases, meaning that the surface is thermodynamically rough.

To our surprise, in the present work the roughened surface was found to have a KPZ roughness exponent. In the non-equilibrium steady state, the roughness exponent $\alpha$ determines the universality class for a 2D growing surface (Eq. (2)). In Fig. 1c the surface widths are scaled by $L^{\alpha }$ and exhibit good agreement with the KPZ roughness exponent ($\alpha \approx 0.385$). Herein, the KPZ roughening point is designated as $\Delta \mu _\text{KPZ}^{(001)}= 0.55 \varepsilon$, while the end of the KPZ rough surface is designated as $\Delta \mu _\text{KtoB}^{(001)} = 2.0 \varepsilon$.

In Fig. 2a–c, images acquired at $4 \times 10^8$ MCS/site are shown for $\Delta \mu /\varepsilon = 0.8$, 1.4 and 2.6, respectively. From Fig. 2a,b, it is evident that poly-nucleated multilayer islands appeared on the (001) surface and the perimeter of each island is also apparent. At $\Delta \mu /\varepsilon = 0.8$ (Fig. 2a), *island-on-island* structures can be seen, otherwise referred to as multilayer islands^57^, having distorted square morphologies. The side view presented in Fig. 2b also clearly indicates an island-on-island structure associated with $\Delta \mu /\varepsilon = 1.4$. These islands are known to coalesce to complete the growth layer^24–26^, and so a self-affine surface exhibiting KPZ roughness was formed based on the island-on-island structure.

It should be noted that this KPZ rough surface represents a type of faceted rough surface that is atomically smooth but thermodynamically rough. The authors previously proposed the new concept of a so-called faceted rough surface and provided numerical evidence related to kinetic roughening^1^ based on evaluating the surface roughness of surface systems between the atomic and mesoscopic length scales. A faceted rough surface is defined as being atomically smooth but thermodynamically rough, even though such surfaces grow via a 2D poly-nucleation process. Our prior work^1^ using the Monte Carlo method demonstrated that an inclined surface meeting the criteria will be thermodynamically rough with a roughness exponent of $\alpha =0.60$ in the non-equilibrium steady state. This result provided evidence for the possibility of a large roughness exponent.

**Figure 3:**
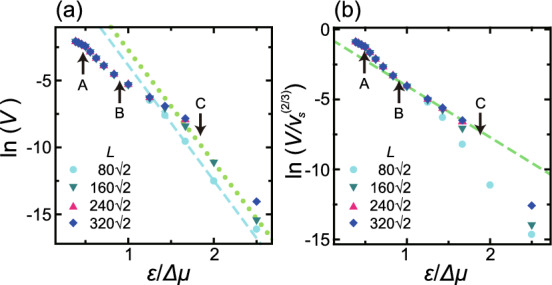
Surface growth velocity data for a 2D nucleation process. The points A, B and C indicate $\Delta \mu _\text{KtoB}^{(001)}$, $\Delta \mu _{kr}^{(001)}$ and $\Delta \mu _\text{KPZ}^{(001)}$, respectively. (**a**) Dotted line: $y=-8.45 x+5.72$, where $y=\ln (V)$ and $x=1/\Delta \mu$. Dashed line: $y=-8.62x+4.76$. (**b**) Dashed line $y=-3.67 x -0.373$, where $y=\ln (V/v_s^{2/3})$. $p=0$. $k_\text{B}T/\varepsilon = 0.4$.

$\Delta \mu _\text{KPZ}^{(001)}$ is also a crossover point between the single and poly-nucleation growth processes. For small $\Delta \mu$ values, *V* will be proportional to the single nucleation rate per area, $I_n \propto \exp (-G^*/k_\text{B}T)$, according to the relationship $V=L^2 I_n$. Here, $G^*$ is the free energy change for the formation of a critical nucleus. Based on the thermodynamics of a 2D island (see the “Analysis of 2D single nucleation” subsection in the Methods section), $G^*/k_\text{B}T$ can be expressed as $G^*/k_\text{B}T=\gamma _{s,\text{total}}^2/(4S k_\text{B}T\Delta \mu ) \equiv g^*/\Delta \mu$ (Eqs. (12) and (13)), where $\gamma _{s,\text{total}}$ is the total step free energy at the perimeter of the 2D equilibrium crystal shape with the Lagrange multiplier being 1 and *S* is the corresponding area^58^. In this work, $\gamma _{s,\text{total}}$ and *S* were calculated based on the 2D Ising model using the imaginary path-weight random walk method^59,60^. Then, $\gamma _{s,\text{total}}a/\varepsilon =6.441$ and $S/a^2=3.001$ were determined at $k_\text{B}T/\varepsilon =0.4$. On this basis, a value of $g^*/\varepsilon =8.640$ was obtained.

Figure 3 plots $\ln (V)$ as a function of $\varepsilon /\Delta \mu$ and $\ln (V/v_s^{2/3})$ as a function of $\varepsilon /\Delta \mu$ based on the Monte Carlo results in Fig. 1a, where $v_s$ is the step velocity (Eq. (15) in the Methods section). For $\Delta \mu < \Delta \mu _\text{KPZ}^{(001)}$, the slopes of the lines obtained using different system sizes are in good agreement and are close to the value of 8.640 calculated using the Ising model. For larger $\Delta \mu$, the logarithm of $V/v_s^{2/3}$ was plotted against $1/\Delta \mu$ (Fig. 3b) because

7 $$\begin{aligned} V \propto (v_s)^{2/3} I_n^{1/3} \end{aligned}$$

for 2D poly-nucleation^25,26^.

The Monte Carlo results obtained for $0.55<\Delta \mu /\varepsilon <1.2$ form a suitably straight line for $L=320 \sqrt{2}$, $240 \sqrt{2}$ and $160 \sqrt{2}$. The least squares fit to these values gave a slope of $-3.67$, the absolute value of which is larger than the expected value of $g^*/3 = 2.88$ based on 2D poly-nucleation theory^25,26^ Eq. (7). In contrast, in the case of $1.2<\Delta \mu /\varepsilon$, the Monte Carlo data deviate from a straight line. Here it is important to recall that $\Delta \mu _{kr}^{(001)}= 1.15 \varepsilon$^39^ for a relatively high growth velocity, *V* (Fig. 1a).

**Figure 4:**
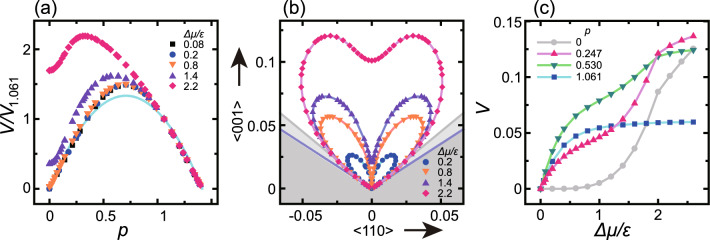
The effects of slope, *p* and $\Delta \mu$ on surface growth velocity. (**a**) The surface growth velocity scaled by $V_{1.061}$ (the surface growth velocity at $p=1.061$) as a function of the slope value. Line: Eq. (10). Symbols: $L= 240 \sqrt{2} a$, $160 \sqrt{2} a$ and $80 \sqrt{2} a$ with $a=1$. Note that *V* is independent of system size. (**b**) A polar graph of surface velocity normal to the inclined surface, $V_n= V/\sqrt{g}$, where $g= 1+p_x^2+p_y^2$. Taking angle $\theta =0$ as the $\langle 001 \rangle$ direction, $V_n$ are plotted from the origin to the normal direction of the surface between the $\langle \bar{1}\bar{1}1 \rangle$ ($-54.74^{\circ }$) and $\langle 111\rangle$ (54.74$^{\circ }$) directions. Dark shaded area: surface orientations less than −54.74$^{\circ }$ and larger than 54.74$^{\circ }$. Light shaded area: terrace-step-kink (TSK) regions with $0.9<|p|$. $L= 240 \sqrt{2}$. (**c**) Surface growth velocity as a function of $\Delta \mu$ for several slopes. $L= 160 \sqrt{2} a$ with $a=1$. $k_\text{B}T/\varepsilon =0.4$. $\Delta \mu _{cr}/\varepsilon = 0.3$.

As is evident from Fig. 1b, for $2.0 \le \Delta \mu /\varepsilon$, the surface approaches BKT roughness as $\Delta \mu$ is increased. In addition, at $\Delta \mu /\varepsilon = 2.6$ (Fig. 2c), island-on-island structures are still seen but the size of the islands decreases and the side view of the surface indicates a greater number of fine irregularities. Furthermore, the surface velocity in this region increases linearly as $\Delta \mu$ increases (Fig. 1a). Based on the results concerning this structure and the surface growth velocity data, the surface can be said to be kinetically, atomically and thermodynamically rough.

For large $\Delta \mu$, various types of kinetic roughening are known to occur in the crystal growth field^22–26,57^. One kinetic roughening point with the classical criteria^22^, $\Delta \mu _c$, is defined as $\pi \gamma _s^2/(3k_\text{B}T\Delta \mu _c) \sim g^*/(3 \Delta \mu _c) \sim 1$^25^ or $g^*/\Delta \mu _c \sim 1$^26^. By substituting $g^*$ of the Monte Carlo result in the equation, we have $\Delta \mu _c/\varepsilon \sim 2.88$ or 8.64, which is a large value compared with the Monte Carlo result of $\Delta \mu _\text{KtoB}^{(001)} =2.0$ at which adhesive growth starts for $\Delta \mu _\text{KtoB}^{(001)} <\Delta \mu$.

Another criterion for $\Delta \mu _c$ is obtained from the zeros of the non-equilibrium step free energy^23^. Leaving aside whether non-equilibrium step free energy is well defined, the zeros of the step free energy explain why the surface is atomically and thermodynamically rough, and why the surface steps are not clear. We estimated $\Delta \mu _c/\varepsilon$ for the Cuppen et al. results for the present case using Fig. 2 in Ref.^23^, giving a value of 1.3, where $\phi /k_\text{B}T$ is 2.5, and $\Delta \mu _c/k_\text{B}T\sim 3.3$. $\Delta \mu _c/\varepsilon \sim 1.3$ is small compared with the Monte Carlo result of $\Delta \mu _\text{KtoB}^{(001)} =2.0$.

It should be noted that the size of the critical nucleus is 1 at $\Delta \mu /\varepsilon =2.0$ if we assume that the island shape is square (Eq. (14) in the Methods section). As stated by Saito^25^, another criterion for $\Delta \mu _c$ is that the size of the critical nucleus becomes sufficiently small. We may therefore adopt “the size of the critical nucleus is one” as the criterion for $\Delta \mu _\text{KtoB}^{(001)}$. According to the 2D single nucleation theory (see the “Analysis of 2D single nucleation” subsection in the Methods section), the quantities at equilibrium are $\Delta \mu$ and $\hat{\gamma }_{s,\text{total}}$, where $\hat{\gamma }_{s,\text{total}}$ is the total step free energy around the 2D island. The step free energy at equilibrium should be calculated in the long step length limit to correctly take into consideration the entropy for the zig-zag structure of a step. However, when the size of the critical nucleus becomes 2 or 1, the entropy for the zig-zag structure is almost excluded because the step is too short to take a zig-zag structure. Since the lattice structure is simple cubic, the shape of the nucleus can be assumed to be a square with straight sides for a small nucleus, resulting from the finite size effect. Then, from Eq. (14) in the Methods section, the size of the critical nucleus $\lambda _c$ is given by $\lambda _c=2\varepsilon /\Delta \mu$. For $1.0<\Delta \mu /\varepsilon <2.0$, the critical size of a square on a (001) terrace is less than 2 but larger than 1. This means that on average a single atom on the (001) terrace detaches. For $2.0<\Delta \mu /\varepsilon$, the critical size of a square on a (001) terrace is less than 1. Hence, on average less than a single atom detaches from the (001) terrace. Therefore, we adopt “the size of the critical nucleus is one” as the criterion for $\Delta \mu _\text{KtoB}^{(001)}$. We will return to this point in the Discussion section.

#### AKPZ criteria

Here it is helpful to examine the extent of agreement between the Monte Carlo results and the KPZ equation. The crossover from BKT roughness to KPZ roughness on a surface can be discussed using the arguments proposed in Refs.^52,61^. The relationship between the surface velocity and the fluctuation width was discussed by Wolf^61^ using the renormalization group method based on the anisotropic KPZ (AKPZ) model. The values of $\lambda _{\tilde{x}}$ and $\lambda _{\tilde{y}}$ are given by

8 $$\begin{aligned} \lambda _{\tilde{x}}=\partial ^2 V/\partial p^2, \quad \lambda _{\tilde{y}}=(\partial V/\partial p)/p, \end{aligned}$$

where *p* is the surface slope in the $\tilde{x}$ direction for an inclined surface. Wolf^61^ reported the criteria for the classification of surface width as follows:

9 $$\begin{aligned} \lambda _{\tilde{x}} \lambda _{\tilde{y}} > 0{} & {} \quad W \propto L^\alpha \ (\text{algebraic} \ \text{rough}) \nonumber \\ \lambda _{\tilde{x}} \lambda _{\tilde{y}} \le 0{} & {} \quad \alpha =0, \ W^2 \propto \ln L. \end{aligned}$$

**Figure 5:**
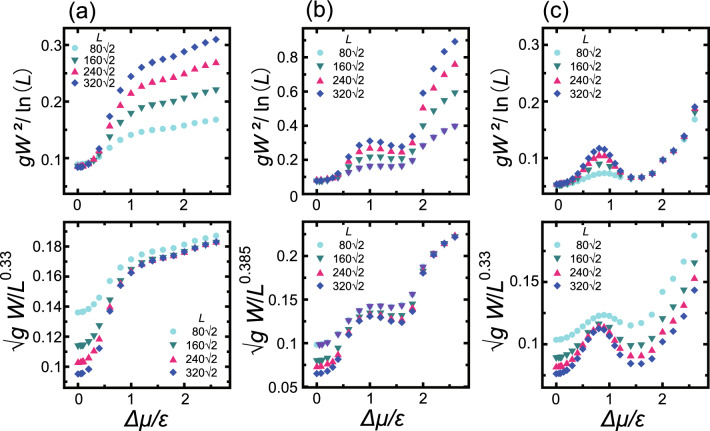
Scaled surface widths as functions of the driving force with (**a**) $p=3\sqrt{2}/4 \approx 1.061$, (**b**) $p=3\sqrt{2}/8 \approx 0.530$ and (**c**) $p = 7\sqrt{2}/40 \approx 0.247$. The upper subfigures show $gW^2$ scaled by $\ln L$. The lower subfigures show $\sqrt{g}W$ scaled by $L^{\alpha }$. $k_\text{B}T/\varepsilon =0.4$. $\Delta \mu _{cr}/\varepsilon = 0.3$.

**Figure 6:**
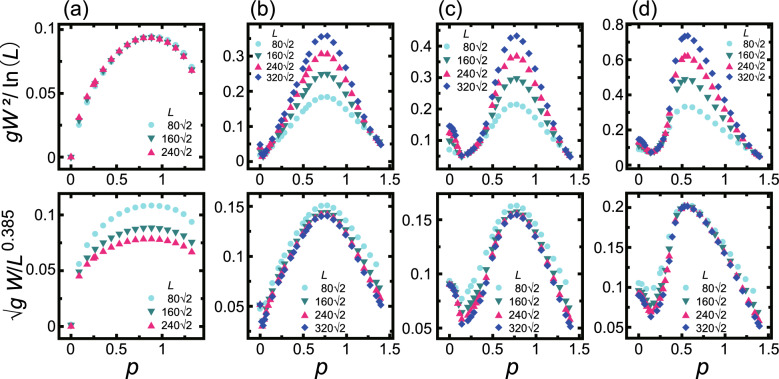
Scaled surface widths as functions of the slope. (**a**) $\Delta \mu /\varepsilon =0.2$. (**b**) $\Delta \mu /\varepsilon =0.8$. (**c**) $\Delta \mu /\varepsilon =1.4$. (**d**) $\Delta \mu /\varepsilon =2.2$. The upper subfigures show $gW^2$ scaled by $\ln L$. The lower subfigures show $\sqrt{g}W$ scaled by $L^{\alpha }$ with a KPZ roughness exponent of $\alpha \approx 0.385$. $k_\text{B}T/\varepsilon =0.4$. $\Delta \mu _{cr}/\varepsilon = 0.3$.

**Figure 7:**
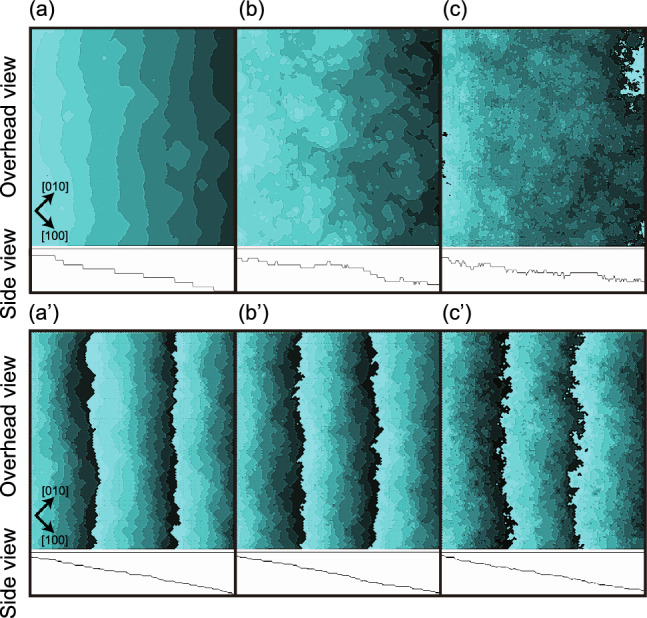
Images of simulated inclined surfaces. $k_\text{B}T/\varepsilon =0.4$. $\Delta \mu _{cr}/\varepsilon = 0.3$. (**a**) and (**a′**) $\Delta \mu /\varepsilon =0.8$. (**b**) and (**b′**) $\Delta \mu /\varepsilon =1.4$. (**c**) and (**c′**) $\Delta \mu /\varepsilon =2.2$. (**a**–**c**) $N_\text{step}=8$. (**a**) $L=320 \sqrt{2}$. (**b**) and (**c**) $L=240 \sqrt{2}$. (**a′**), (**b′**) and (**c′**) $p=7\sqrt{2}/40\approx 0.247$ surface. $N_\text{step} = 28$. $L = 80 \times \sqrt{2}$.

For the limit $p \rightarrow 0$, the surface growth velocity on an inclined surface can be expressed as $V \approx v_s p$ for $\Delta \mu <\Delta \mu _\text{KPZ}^{(001)}$ (Fig. 4a,b). In the case of this $\Delta \mu$ range, the surface grows based on a TSK process. In contrast, for $\Delta \mu _\text{KPZ}^{(001)}<\Delta \mu <\Delta \mu _\text{KtoB}^{(001)}$, islands are frequently formed on the (001) terraces. Using a magnified version of Fig. 4a, we confirmed that $V = V_0+ c_{p} p^2+ {\mathscr O}(p^3)$ for $p \rightarrow 0$, where $c_{p}$ is a positive coefficient at $\Delta \mu /\varepsilon =0.8$ with $L=320 \sqrt{2}$. Note that this effect of the slope on *V* in the vicinity of $p=0$ is revisited in the subsection titled “Kinetic shape changes of a crystallite.”

For $\Delta \mu _\text{KPZ}^{(001)}<\Delta \mu <\Delta \mu _\text{KtoB}^{(001)}$, $\partial ^2 V/\partial p^2>0$ and $(\partial V/\partial p) /p >0$ near $p=0$, giving a coefficient value of $\lambda _x \lambda _y > 0$. Hence, the surface should be algebraically rough and the Monte Carlo data also show KPZ roughening on the surface.

In the case of $\Delta \mu _\text{KtoB}^{(001)}< \Delta \mu$, we have $\lambda _x \lambda _y > 0$ for the same reason as in the case of $\Delta \mu _\text{KPZ}^{(001)}<\Delta \mu <\Delta \mu _\text{KtoB}^{(001)}$. Consequently, the surface should be algebraically rough but the Monte Carlo results suggest BKT roughening. Therefore, we conclude that the AKPZ criteria (Eq. (9)) are partly consistent with the Monte Carlo results for a (001) surface.

### Inclined surfaces

This section clarifies the difference in surface roughness between a TSK^41,42^ model surface and a surface with terrace roughness for inclined surfaces. In the ideal TSK model, an inclined surface consists of a train of elementary steps having unit heights with no islands or negative islands (that is, clusters of adholes) on the terraces.

Figure 5 summarizes the effect of $\Delta \mu$ on the scaled surface width for several surface slopes and system sizes at a temperature of $k_\text{B}T/\varepsilon =0.4$. Here, the upper subfigures show the squared surface widths scaled by $\ln L$, while the lower subfigures present the surface widths scaled by $L^{\alpha }$. In Fig. 6, the calculated scaled surface widths for several $\Delta \mu$ and system sizes at a temperature $k_\text{B}T/\varepsilon =0.4$ are plotted as functions of slope. The upper subfigures show the squared surface widths scaled by $\ln L$, while the lower subfigures show the surface width scaled by $L^{\alpha }$ with $\alpha =0.385$ (that is, the value of the KPZ roughness exponent).

The logarithmic divergence of $gW^2$ for an inclined surface at equilibrium is well established^4,5,18,19,39^. Here, $g= 1+p_x^2+ p_y^2$ is the first fundamental quantity in the differential geometry^10,55^. For all slopes near equilibrium, the surface with $\Delta \mu /\varepsilon \le 0.3$ exhibits BKT roughening (see Figs. 5 and 6a). Hence, the present work confirms that the crossover point between BKT and KPZ roughening of an inclined surfaces is $\Delta \mu _{cr}/\varepsilon = 0.3$.

#### Surfaces with almost ideal TSK structures

Hereafter, we consider an inclined surface for which $\Delta \mu _{cr}< \Delta \mu$. As is evident from Fig. 4a, in the case of large surface slopes ($0.9<p$) near the (111) surface, the surface growth velocities are in good agreement with one another because the surfaces have almost the ideal TSK structure due to the RSOS restriction^39,43,62^. Figure 4c indicates the effect of $\Delta \mu$ on *V* and demonstrates that *V* for $p=1.061$ plateaus at $0.8 \le \Delta \mu /\varepsilon$. Physically, surface growth occurs via the attachment and detachment of the atoms (here represented as cubes or growth units) at the step edges.

The lower subfigure in Fig. 5a demonstrates that, at $p=1.061$, a stepped surface without terrace islands becomes algebraically rough at $\Delta \mu _{cr}< \Delta \mu$. Here, the roughness exponent is $\alpha = 0.33$ and so is slightly smaller than the expected value for a KPZ roughened surface. From Fig. 6b–d, it is apparent from the surface widths for surfaces with $0.9<p<1.25$ that the surfaces are algebraically rough. In addition, the roughness exponent appears to gradually decrease as *p* increases.

At this point, it is helpful to ascertain agreement with the AKPZ criteria in Eq. (9). Since $\partial (V/V_{1.601})/\partial p <0$ and $\partial ^2 V/V_{1.601}/\partial p^2 < 0$, $\lambda _{\tilde{x}} \lambda _{\tilde{y}} > 0$ based on Eq. (9). Hence, the surfaces should be algebraically rough, which is consistent with the Monte Carlo results for $0.9<p$. For the limit $p \rightarrow \sqrt{2}$, the present numerical results confirm that $\partial ^2 (V/V_{1.601})/\partial p^2 \rightarrow 0$ and that $V= v_s^\text{neg}(\sqrt{2}-p)$, where $v_s^\text{neg}$ is the step velocity for a negative step, meaning a step associated with a (111) terrace. These results demonstrate that the contribution of the nonlinear terms in the KPZ or AKPZ equation are reduced as *p* approaches $\sqrt{2}$.

On this basis, we conclude that the effects of the slope and $\Delta \mu$ on the surface width for $0.9<p$ as obtained using the Monte Carlo method are consistent with the KPZ or AKPZ criteria.

#### Kinetic shape changes of a crystallite

Figure 4c presents results for surfaces with $p=0.247$ and 0.530 and shows that *V* increases steeply for $\Delta \mu _{kr}^{(001)}<\Delta \mu$ as $\Delta \mu$ increases, except for the surface for which $p=1.061$. This steep increase in *V* (other than the above exception) provides evidence that island formation on (001) terraces resulting from the 2D nucleation process causes a steep increase in *V*, as is also the case for $p=0$.

When assessing crystal growth, it is interesting that the anisotropy with respect to *V* for $\Delta \mu < \Delta \mu _\text{KPZ}^{(001)}$ is large compared with that in the corresponding Wulff figure^63^ showing the polar graph of the surface tension. This can be seen from Fig. 4b. The significant anisotropy of *V* indicates that the crystallite grows such that it has a wider (001) facet compared with the equilibrium shape for $\Delta \mu < \Delta \mu _\text{KPZ}^{(001)}$.

This effect occurs because there are two kinds of steps around $p \sim 0.7$ associated with two kinds of terraces: the (001) and (111) terraces. As noted in the previous section, steps with (111) terraces and (001) side surfaces can be considered as negative steps. In this scenario, steps with small *p* values will grow to the right (e.g. see Fig. 7), whereas negative steps with large *p* values will grow to the left. For *p* values of 0.5–0.8, a surface having a mixture of steps including negative steps will grow in both directions (see the Supplementary Movie 1 S1). In the case of $\Delta \mu <\Delta \mu _\text{KPZ}^{(001)}$, the surface velocity, *V*, can be written as

10 $$\begin{aligned} V=\,\,& {} v_s p\ w^{(001)} +v_s^\text{neg} (\sqrt{2}-p) w^{(111)}, \nonumber \\{} & {} w^{(001)}=(\sqrt{2}-p)/\sqrt{2}, \ w^{(111)}= p/\sqrt{2}, \end{aligned}$$

where $w^{(001)}$ and $w^{(111)}$ are the statistical weights for the number of steps determined by the surface slope, *p*. Because $v_s$ is approximately equivalent to $v_s^\text{neg}$ (see Eq. (15)) for $\Delta \mu < \Delta \mu _\text{KPZ}^{(001)}$, the slope dependence of *V* is almost symmetrical along with $p=1/\sqrt{2}$ (Fig. 4a). Figure 4a plots the line obtained from Eq. (10) with $V_{1.061}=(v_s+v_s^\text{neg})3/(8\sqrt{2})$ and this line is seen to be in good agreement around both limits $p \rightarrow 0$ and $p \rightarrow \sqrt{2}$. It should also be noted that the values obtained using the Monte Carlo method for $p \sim 0.7$ are higher than those produced by Eq. (10).

For $\Delta \mu _\text{KPZ}^{(001)}<\Delta \mu$, because $\partial V/\partial p =0$ at $p=0$, the planar shape on the (001) surface becomes unstable in conjunction with infinitesimally small fluctuations in the slope. Hence, a kinetic version of the faceting transition is expected to occur at $\Delta \mu _\text{KPZ}^{(001)}$. For $\Delta \mu _\text{KPZ}^{(001)}<\Delta \mu <\Delta \mu _{kr}^{(001)}$, *V* continues to exhibit a high degree of anisotropy. Hence, the (001) surface should not be planar but rather should exhibit some small degree of curvature. For $\Delta \mu _{kr}^{(001)}<\Delta \mu$, the anisotropy in *V* is drastically reduced in the vicinity of the (001) surface. In particular, in the region $\Delta \mu _\text{KtoB}^{(001)}<\Delta \mu$, the concentration of adatoms on the (001) terraces is so large that the elementary step as the edge of the (001) terrace cannot be well defined (see Fig. 7c,c’), similar to the behavior of a surface with $T_\text{R}<T$.

#### Nonlinear effect

For $p<0.4$, the roughness change is complex due to the interplay between multilayered islands and steps. Near $p=0$, the surfaces are algebraically rough and $\sqrt{g}W$ decreases as *p* increases, as shown in Fig. 6b–d. With increases in *p*, a transition to a BKT roughened surfaces occurs near $p\sim 0.05$ that is dependent on $\Delta \mu$. Here, the crossover point for *p* is denoted as $p_\text{KtoB}^{(p<0.4)}$. For $\Delta \mu /\varepsilon =0.8$, 1.4 and 2.2, $p_\text{KtoB}^{(p<0.4)}\approx 0.017$, 0.051 and 0.073, respectively. In the vicinity of $p=0.3$, there is another transition to an algebraically roughened surface and this crossover point is denoted as $p_\text{BtoK}^{(p<0.4)}$. For $\Delta \mu /\varepsilon =0.8$, 1.4 and 2.2, $p_\text{BtoK}^{(p<0.4)}=0.11$, 0.28 and 0.30, respectively. Both $p_\text{KtoB}^{(p<0.4)}$ and $p_\text{BtoK}^{(p<0.4)}$ increase as $\Delta \mu$ increases.

**Table 1:** Characteristic driving forces and slopes ($L/(\sqrt{2}a)\ge 240$, $a=1$) for $k_\text{B}T/\varepsilon =0.4$.

Symbol	Value$/\epsilon$	*p*	Description
(001)	Surface		
$\Delta \mu _{\textrm{KPZ}}^{(001)}$	0.55	0	Smooth to KPZ rough surface transition point.
			For $\Delta \mu < \Delta \mu _{\textrm{KPZ}}^{(001)}$, $V=v_s p$ ($p \rightarrow 0$); whereas for $\Delta \mu _{\textrm{KPZ}}^{(001)}< \Delta \mu$, $V=V_0+ c_{p} p^2$ ($0<c_p$, $p \rightarrow 0$). This change is explained by the AKPZ criteria.
$\Delta \mu _{kr}^{(001)}$^39^	1.15	0	For $\Delta \mu _{kr}^{(001)}< \Delta \mu$, $V_0$ becomes relatively large.
$\Delta \mu _{\textrm{KtoB}}^{(001)}$	2.0	0	Crossover point between a 2D poly-nucleation process (a KPZ roughened surface) and an adhesive growth process with kinetic and atomic roughening (a BKT roughened surface).
Inclined	Surface		
$\Delta \mu _{cr}$	0.3	$0<p<\sqrt{2}$	Crossover point from a BKT roughened to algebraic or KPZ roughened surface^39^.
$\Delta \mu _{\textrm{KtoB}}^{(p<0.4)}$	1.15	0.247	Crossover point from a KPZ roughened to BKT roughened surface. This crossover is explained by the competition between the step growth velocity and 2D nucleation rate on terraces.
$p_{\textrm{KtoB}}^{(p<0.4)}$	Dependent on $\Delta \mu$	0–0.17	Crossover point from KPZ or algebraic roughened to BKT roughened surface. This crossover is explained by the AKPZ criteria.
$p_{\textrm{BtoK}}^{(p<0.4)}$	Dependent on $\Delta \mu$	0.1–0.4	Crossover point from BKT to algebraic or KPZ roughened surface. This crossover is explained by the competition between the step growth velocity and 2D nucleation rate on terraces.

Here, it is helpful to assess the level of consistency between Monte Carlo results and the AKPZ criteria. Using the data in Fig. 4a, the inflection point with respect to *p* was calculated based on the Monte Carlo data. This process gave 0.056, 0.17 and 0.14 for $\Delta \mu /\varepsilon = 0.8$, 1.4 and 2.2, respectively and these inflection points are close to those for $p_\text{KtoB}^{(p<0.4)}$. That is, when $p<p_\text{KtoB}^{(p<0.4)}$, $\partial ^2 V/\partial p^2>0$. On the basis of the criteria given by Eq. (9), the surface should therefore be algebraically rough. In contrast, if $p_\text{KtoB}^{(p<0.4)}<p <0.4$, $\partial ^2 V/\partial p^2<0$ and Eq. (9) suggests that the surface should exhibit BKT roughening. Figure 7a–c demonstrate the coalescence of terrace islands to steps and this process enhances the step fluctuations for small values of *p* ($N_\text{step} =8$). Therefore, we conclude that the nonlinear effect associated with surface growth makes the inclined surface near $p=0$ algebraically rough.

Physically, the inflection point can be explained by an effect in which the inclined steps hinder the formation and free growth of multilayered islands. Figures 7a′–c′ present images of the surfaces for $p=0.247$ ($N_\text{step} =28$) and it is apparent that these surfaces appear different from those in (a), (b) and (c). In Fig. 7 a′–c′, fewer multilayered islands appear than in Fig. 2 and so it is evident that the KPZ structure was changed to a BKT structure.

In the case of $p_\text{BtoK}^{(p<0.4)}<p$, the transition from BKT roughened to algebraically roughened cannot be explained by the KPZ criteria. The following subsection discusses the origin of this crossover point.

#### Competition between step growth velocity and nucleation rate on the terrace

From Fig. 5c, it is apparent that, for $\Delta \mu /\varepsilon \sim 1.5$ at $p=0.247$, the surface width becomes BKT rough. A peak around $\Delta \mu /\varepsilon \sim 0.8$ is also apparent. In our previous work^39^, the peak was thought to be related to a kinetic roughening. However, from the extensive calculations in this study, that conclusion was found to be incorrect. In Fig. 7a′, the surface structure at $\Delta \mu /\varepsilon =0.8$ is shown and this surface appears to be a TSK-type stepped surface. Few adatoms or adholes are seen on terraces in this area. Rather, as shown in the case of $p=1.061$, the TSK like surface crossovers from BKT rough to a power law rough for $\Delta \mu _{cr}< \Delta \mu$.

While, for the surface structure at $\Delta \mu /\varepsilon =1.4$, which is presented in Fig. 7b′, the elementary steps show numerous overhang structures and there are small numbers of islands or negative islands on the terraces. The poly-nucleated clusters on the terraces merge with growing steps, and then the step edges have generated overhang structures. Because islands having different heights or negative islands cannot merge completely with steps, the higher islands or lower negative islands act as obstacles to the growing steps. In this manner, fluctuations of the steps are reduced by the multi-height islands or negative islands. For $\Delta \mu _{kr}^{(001)} <\Delta \mu$, there is a non-negligible reduction in step fluctuations that produces a BKT roughened surface. As a result, the surface width decreases to form a peak as seen in Fig. 5c. We denote this crossover point for $\Delta \mu$ as $\Delta \mu _\text{KtoB}^{(p<0.4)}$ and note that $\Delta \mu _\text{KtoB}^{(p<0.4)}$ is close to $\Delta \mu _{kr}^{(001)}$ when $p=0.247$.

## Discussion

In the case that of an inclined surface for which the temperature is higher than $k_\text{B}T/\varepsilon =0.4$, the crossover point $\Delta \mu _{cr}$ becomes larger^39^. Hence, for $k_\text{B}T/\varepsilon =0.63$ and 1.7, $\Delta \mu _{cr}/\varepsilon$ becomes 0.5 and 1.2, respectively^39^. The population of adatoms on the (001) terraces will therefore be larger at higher temperatures and these adatoms will partially block the step fluctuations that generate surface BKT roughening.

In contrast, $\Delta \mu _\text{KPZ}^{(001)}$ decreases rapidly as temperature increases. Since the step free energy at equilibrium decreases as the temperature increases, 2D nuclei are formed more frequently, which leads to a crossover to poly nucleation easily. $\Delta \mu _\text{KPZ}^{(001)}$ becomes almost zero at $k_\text{B}T/\varepsilon \sim 0.9$ in our preliminary simulations.

Figures 4a,b reproduce qualitatively the characteristics of the angle dependence of the growth rate shown in Fig. 4 in the paper of Van Veenendaal et al.^22^, although the models are different from each other. At small $\Delta \mu$ (Fig. 4 a in Ref. [22]), the angle dependence of the surface growth rate has a cusp singularity at $p=0$ for $\phi /k_\text{B}T=2$ and $\Delta \mu /k_\text{B}T=1$ ($k_\text{B}T/\varepsilon = 0.5$ and $\Delta \mu /\varepsilon =0.5$). For large $\Delta \mu$ (Fig. 4 c in Ref. [22]), where $\phi /k_\text{B}T=2$ and $\Delta \mu /k_\text{B}T=3$, the angle dependence of the surface growth rate is parabolic at $p=0$. These agreements between the present results and Van Veenendaal et al.’s results around $p=0$ indicate that the growth shape change of a crystalline near the (001) facet is a universal phenomenon associated with KPZ roughening.

On the other hand, for $\Delta \mu _c$ for the (001) surface, our preliminary results were complex. If we adopt “the size of the critical nucleus is one” as the criterion for $\Delta \mu _\text{KtoB}^{(001)}$, $\Delta \mu _\text{KtoB}^{(001)}$ does not depend on temperature if $\varepsilon$ does not change. Referring to Fig. 2 in Ref.^23^, $\Delta \mu _c$ decreases almost linearly from the value for $k_\text{B}T/\varepsilon =0.5$ to zero at about the $T_c$ of the 2D nn Ising model. From our preliminary Monte Carlo results, there is a possibility that $\Delta \mu _\text{KtoB}^{(001)}$ may be different from $\Delta \mu _c$. For the surface growth velocity, $\partial V/\partial \Delta \mu$ seems to change discontinuously around $\Delta \mu /\varepsilon =2.0$ for several temperatures up to $k_\text{B}T/\varepsilon =1.4$, which is lower than $T_\text{R}^{(001)}$ but higher than $T_c$ of the 2D nn Ising model. In addition, another characteristic point $\Delta \mu _{kr}^{(001)}$ also decreases as temperature increases. At $k_\text{B}T/\varepsilon =0.83$, $\Delta \mu _{kr}^{(001)}/\varepsilon$ becomes about 0.2 with $\partial ^2 V/\partial \Delta \mu ^2|_{\Delta \mu /\varepsilon =0.2} >0$. While for $k_\text{B}T/\varepsilon =1.4$, $\Delta \mu _{kr}^{(001)}/\varepsilon$ is zero and $\partial ^2 V/\partial \Delta \mu ^2|_{\Delta \mu /\varepsilon =0}$ is also zero. The surface is BKT rough at the region $0 \le \Delta \mu /\varepsilon <2.0$.

These preliminary results suggest that the interplay between KPZ roughening and BKT roughening around $T_\text{R}^{(001)}$ may occur. In this manner, the temperature dependences of $\Delta \mu _c$ and $\Delta \mu _\text{KtoB}^{(001)}$ are still open questions. Further studies are expected.

## Conclusions

The following are the conclusions obtained from the present study of (001) surfaces.

- Monte Carlo results for $0 \le \Delta \mu < \Delta \mu _\text{KPZ}^{(001)}$ show that the surface remains smooth during a single nucleation process. In contrast, in the case of $\Delta \mu _\text{KPZ} ^{(001)}\le \Delta \mu < \Delta \mu _\text{KtoB}^{(001)}$, the surface undergoes KPZ roughening and grows via a 2D poly-nucleation process. The multilayer islands were found to be essential for the formation of the self-affine surface structure
- For $\Delta \mu _\text{KtoB}^{(001)} \le \Delta \mu$, the surface undergoes BKT roughening and is also kinetically and atomically rough with adhesive growth. The steps on the surface are difficult to define, similar to the case of a rough surface at temperatures defined by $T_\text{R}<T$.

The conclusions for inclined surfaces are as follows.

- In the case of $0<p<\sqrt{2}$, an inclined surface will exhibit BKT roughening for $\Delta \mu < \Delta \mu _{cr}$, where $\Delta \mu _{cr}$^39^ is a crossover point between BKT and algebraically rough surfaces
- The roughness of inclined surfaces varies in a complex manner depending on the values of $\Delta \mu$ and slope, *p*, due to the interplay between step growth and the formation of multilayered islands on (001) terraces. The crossover points between BKT and algebraically rough surfaces are summarized in Table 1
- The surface growth velocity, *V*, exhibits greater anisotropy than that associated with surface tension for $\Delta \mu < \Delta \mu _\text{KPZ}^{(001)}$ due to the possibility of two kinds of steps: those with (001) terraces and those with (111) terraces. The growth shape of a crystallite involves a wider facet area than that at equilibrium. For $\Delta \mu _\text{KPZ}^{(001)}< \Delta \mu$, the non-equilibrium KPZ roughening transition induces a kinetic change in the crystallite shape on the nanoscale such that the (001) facets have very slightly curved surfaces. For $\Delta \mu _\text{KtoB}^{(001)}<\Delta \mu$, the anisotropy of *V* is drastically reduced such that the growth shape is expected to be nearly spherical
- The effects of the slope value on *V* and $\sqrt{g}W$ are not equivalent but are approximately similar.

## Methods

### Analysis of 2D single nucleation

To obtain the nucleation barrier for a non-spherical shape, we introduce the scaling parameter $\lambda$. Assuming the shape of the critical nucleus is similar to the 2D equilibrium crystal shape (ECS), $\gamma _{s,\text{total}}$ is defined as the total step free energy of the ECS with the Lagrange multiplier being 1. If *S* is the area corresponding to the ECS, the island formation free energy, *G*, is given by

11 $$\begin{aligned} G= -\lambda ^2 \Delta \mu S + \lambda \gamma _{s,\text{total}}, \quad \hat{S}= \lambda ^2 S, \ \hat{\gamma }_{s,\text{total}} = \lambda \gamma _{s,\text{total}}, \end{aligned}$$

where $\hat{S}$ is the area of the island and $\hat{\gamma }_{s,\text{total}}$ is the total step free energy at the perimeter of the island. In the case that the island is the critical nucleus, $\partial G/\partial \lambda =0$ and we have

12 $$\begin{aligned} \lambda _c= \gamma _{s,\text{total}}/(2S \Delta \mu ), \ G^*=\lambda _c^2 S \Delta \mu . \end{aligned}$$

Since $G^*/k_\text{B}T\equiv g^*/\Delta \mu$, we can write

13 $$\begin{aligned} g^*=\gamma _{s,\text{total}}^2/(4Sk_\text{B}T). \end{aligned}$$

When the shape is a square with side length $\lambda a$, we have $\hat{S}=(\lambda a)^2/a^2$ and $\hat{\gamma }_{s,\text{total}}=4 (\lambda a)(\varepsilon /a)$, which leads to $S=1$ and $\gamma _{s,\text{total}}=4\varepsilon$. Then, we have

14 $$\begin{aligned} G= -\lambda ^2 \Delta \mu + 4 \lambda \varepsilon , \quad \lambda _c=2 \varepsilon /\Delta \mu , \quad G^*=4\varepsilon ^2/\Delta \mu , \quad g^*=4\varepsilon ^2/(k_\text{B}T). \end{aligned}$$

### Estimation of $v_s$

To obtain the explicit form for $v_s$, this work used parameters that provided the best least squares fit to the Monte Carlo results in Fig. 4 (c) for a negative step velocity $v_s^\text{neg}$ at $p=1.061$. Here, a negative step is defined as a step with a (111) terrace and (001) side surface, such that

15 $$\begin{aligned} v_s^\text{neg}& = {} (a_1 x + a_2 x^2+ a_3 x^3 + a_4 x^4 + a_5 x^5 + a_6 x^6)/(\sqrt{2}-p),\nonumber \\{} & {} x=\Delta \mu /\varepsilon , \ p =3/(2\sqrt{2}) \approx 1.061, \nonumber \\{} & {} a_1= 0.15676,\ a_2 = -0.19464, \ a_3= 0.13698, \ a_4 = -0.055575, \ a_5= 0.012133,\ a_6 = -0.0011059. \end{aligned}$$

The $v_s$ value at $p=0.09$ was confirmed to equal $v_s^\text{neg}$ at $p=1.061$ within a difference of 5%. The results obtained with $p=1.061$ were employed to determine $v_s$ because there was a lack of nucleation on the (111) terraces for negative steps due to the RSOS restriction.

### Supplementary Information

Supplementary Video 1.

## Data Availability

The datasets used and/or analyzed during the current study are available from the corresponding author on reasonable request.

## References

[1] Akutsu N (2021). Faceted-rough surface with disassembling of macrosteps in nucleation-limited crystal growth. Sci. Rep..

[2] Nishinaga T, Sasaoka C, Chernov AA, Sunagawa I (1989). A numerical analysis for the supersaturation distribution around LPE macrostep. Morphology and Growth Unit of Crystals.

[3] Abraham FF, Broughton JQ (1986). Pulsed melting of silicon (111) and (100) surfaces simulated by molecular dynamics. Phys. Rev. Lett..

[4] Weeks JD, Riste T (1980). The roughening transition. Ordering in Strongly Fluctuation Condensed Matter Systems.

[5] van Beijeren H (1977). Exactly solvable model for the roughening transition of a crystal surface. Phys. Rev. Lett..

[6] Berezinskii VL (1971). Destruction of long-range order in one-dimensional and two-dimensional systems having a continuous symmetry group I. Classical Syst. Sov. Phys. JETP.

[7] Kosterlitz JM, Thouless DJ (1973). Ordering, metastability and phase transitions in two-dimensional systems. J. Phys. C.

[8] Jayaprakash C, Saam WF, Teitel S (1983). Roughening and facet formation in crystals. Phys. Rev. Lett..

[9] Rottman C, Wortis M (1984). Statistical mechanics of equilibrium crystal shapes: Interfacial phase diagrams and phase transitions. Phys. Rep..

[10] Akutsu N, Akutsu Y (1987). Roughening, faceting and equilibrium shape of two-dimensional anisotropic interface. I. Thermodynamics of interface fluctuations and geometry of equilibrium crystal shape. J. Phys. Soc. Jpn..

[11] Akutsu N, Akutsu Y (1987). Equilibrium crystal shape: Two dimensions and three dimensions. J. Phys. Soc. Jpn..

[12] Balibar S, Guthmann C, Rolley E (1993). From vicinal to rough crystal surfaces. J. Phys..

[13] Abe H, Saitoh Y, Ueda T, Ogasavara F, Nomura R, Okuda Y, Parshin AY (2006). Facet growth of $$^4$$He crystal induced by acoustic wave. J. Phys. Soc. Japan.

[14] Pavlovska A, Nenaw D (1977). Experimental study of the surface melting of tetrabrommethane. J. Cryst. Growth.

[15] Ohachi T, Taniguchi I (1977). Growth of $$\alpha$$-Ag$$_2$$S and $$\alpha$$-Ag$$_2$$Se single crystals in a solid/vapour system. J. Cryst. Growth.

[16] Ohachi T, Taniguchi I (1983). Roughening transition for the ionic-electronic mixed superioninc conductor $$\alpha$$-Ag$$_2$$S. J. Cryst. Growth.

[17] Akutsu Y, Akutsu N, Yamamoto T (1988). Universal jump of Gaussian curvature at the facet edge of a crystal. Phys. Rev. Lett..

[18] Yamamoto T, Akutsu Y, Akutsu N (1994). Fluctuation of a single step on the Vicinal Surface -Universal and Non-Universal behaviors. J. Phys. Soc. Jpn..

[19] Akutsu Y, Akutsu N, Yamamoto T (1994). Logarithmic step fluctuations in vicinal surface: A Monte Carlo study. J. Phys. Soc. Jpn..

[20] Burton WK, Cabrera N, Frank FC (1951). The growth of crystals and the equilibrium structure of their surfaces. Philos. Trans. R. Soc. Lond. A.

[21] Cahn JW (1960). Theory of crystal growth and interface motion in crystalline materials. Acta Metall..

[22] Van Veenendaal E, van Hoof PJCM, van Suchtelen J, van Enckevort WJP, Bennema P (1998). Kinetic roughening of the Kossel (100) surface: Comparison of classical criteria with Monte Carlo results. Surf. Sci..

[23] Cuppen HM, Meekes H, van Enckevort WJP, Vlieg E, Knops HJF (2004). Nonequilibrium free energy and kinetic roughening of steps on the Kossel(001) surface. Phys. Rev. B.

[24] Ookawa A (1977). Crystal Growth.

[25] Saito Y (1996). Statistical Physics of Crystal Growth.

[26] Pimpinelli A, Villain J (1998). Physics of Crystal Growth.

[27] Uwaha M (2002). Crystal Growth Mechanisms.

[28] Kardar M, Parisi G, Zhang Y-C (1986). Dynamic scaling of growing interfaces. Phys. Rev. Lett..

[29] Vicsek, T. *Surface Disordering: Growth, Roughening, and Phase Transitions*, Jullien, R., Kertesz, J. Meakin, P., Wolf, D. E. (eds) (Nova Science, 1992) 155.

[30] Barabasi AL, Stanley HE (1995). Fractal Concepts in Surface Growth.

[31] Krug J, Spohn H (1991). Solids Far From Equilibrium.

[32] Krug J (1977). Origins of scale invariance in growth processes. Adv. Phys..

[33] Takeuchi KA (2013). Crossover from growing to stationary interfaces in the Kardar–Parisi–Zhang class. Phys. Rev. Lett..

[34] Pagnani A, Parisi G (2015). Numerical estimate of the Karder–Parisi–Zhang universality class in (2+1) dimensions. Phys. Rev. Lett..

[35] Takeuchi KA (2018). An appetizer to modern developments on the Kardar–Parisi–Zhang universality class. Phys. A.

[36] Krim J, Palasantzas G (1995). Experimental observations of self-affine scaling and kinetic roughening at sub-micron length scales. Int. J. Mod. Phys. B.

[37] Gupta I, Mohanty BC (2016). Dynamics of surface evolusion in semiconductor thin films grown from a chemical bath. Sci. Rep..

[38] Almeid RAL, Ferreira SO, Ferraz I, Oliveira TJ (2017). Initial pseudo-steady state & asymptotic KPZ universality in semiconductor on poymer deposition. Sci. Rep..

[39] Akutsu N (2020). ’Crossover from BKT-rough to KPZ-rough surfaces for interface-limited crystal growth/recession. Sci. Rep..

[40] Müller-Krumbhaar H, Binder K (1979). Monte carlo simulation of crystal growth. Monte Carlo Mehtods in Statistical Mechanics.

[41] Gruber EE, Mullins WW (1967). On the theory of anisotropy of crystalline surface tension. J. Phys. Chem. Solids.

[42] Pokrovsky VL, Talapov AL (1979). Ground state, spectrum, and phase diagram of two-dimensional incommensurate crystals. Phys. Rev. Lett..

[43] Akutsu N (2019). Relationship between macrostep height and surface velocity for a reaction-limited crystal growth process. Cryst. Growth Des..

[44] Jayaprakash C, Saam WF (1984). Thermal evolution of crystal shapes: The fcc crystal. Phys. Rev. B.

[45] Yamamoto T, Akutsu Y, Akutsu N (1988). Universal behavior of the equilibrium crystal shape near the facet edge. I. A generalized Terrace–Step–Kink model. J. Phys. Soc. Jpn..

[46] Demange G, Zapolsky H, Patte R, Brunel M (2017). A phase field model for snow crystal gwoth in three dimensions. npj Comutaional Mater..

[47] Kempisty P, Kangawa Y (2019). Evolution of the free energy of the GaN(0001) surface based on first-principles phonon calculations. Phys. Rev. B.

[48] den Nijs M (1985). Corrections to scaling and self-duality in the restricted solid-on-solid model. J. Phys. A Math. Gen..

[49] Akutsu Y (1989). Exact landau free-energy of solvable N-State vertex model. J. Phys. Soc. Jpn..

[50] Bethe HA (1931). Zur theorie der metalle. Zeit. für Physik.

[51] Amar JG, Family F (1990). Phase transition in a restricted solid-onsolid surface-growth model in 2+1 dimensions. Phys. Rev. Lett..

[52] Krug J, Spohn H (1990). Mechanism for rough-to rough transitions in surface growth. Phys. Rev. Lett..

[53] Widom B (2002). Statistical Mechanics: A Concise Introduction for Chemists.

[54] Akutsu N (1992). Equilibrium crystal shape of planar ising antiferromagnets in external fields. J. Phys. Soc. Jpn..

[55] Kreyszig E (1968). Introduction to Differential Geometry and Riemannian Geometry.

[56] Akutsu N (2016). Faceting diagram for sticky steps. AIP Adv..

[57] Markov IV (2003). Crystal Growth for Beginners: Fundamentals of Nucleation, Crystal Growth and Epitaxy.

[58] Akutsu N (2018). Height of a faceted macrostep for sticky steps in a step-faceting zone. Phys. Rev. Mater..

[59] Akutsu Y, Akutsu N (1990). Interface tension, equilibrium crystal shape, and imaginary zeros of partition function: Planar Ising systems. Phys. Rev. Lett..

[60] Akutsu N, Akutsu Y (1999). Statistical mechanical calculation of anisotropic step stiffness of a two-dimensional hexagonal lattice-gas model with next-nearest-neighbor interactkions: application to Si(111) surface. J. Phys. Condens. Matter.

[61] Wolf DE (1991). Kinetic roughening of vicinal surface. Phys. Rev. Lett..

[62] Akutsu N, Akutsu Y (2022). Slope-temperature faceting diagram for macrosteps at equilibrium. Sci. Rep..

[63] Akutsu N (2017). Profile of a faceted macrostep caused by anomalous surface tension. Adv. Condens. Matter Phys..

